# Attention Networks in ADHD Adults after Working Memory Training with a Dual *n*-Back Task

**DOI:** 10.3390/brainsci10100715

**Published:** 2020-10-08

**Authors:** Masashi Dotare, Michel Bader, Sarah K. Mesrobian, Yoshiyuki Asai, Alessandro E. P. Villa, Alessandra Lintas

**Affiliations:** 1 School of Medicine, Yamaguchi University, 1-1-1 Minami-kogushi, Ube, Yamaguchi 755-8505, Japan; 2 Department of Psychiatry -SUPEA, University Hospital of Lausanne, CH-1004 Lausanne, Switzerland; bader_m@bluewin.ch; 3 NeuroHeuristic Research Group, HEC-Lausanne, University of Lausanne, Quartier UNIL-Chamberonne, CH-1015 Lausanne, Switzerland; smesrobian@neuristic.org (S.K.M.); alessandro.villa@unil.ch (A.E.P.V.); 4 Department of Systems Bioinformatics, Graduate School of Medicine, Yamaguchi University, 1-1-1 Minami-Kogushi, Ube, Yamaguchi 755-8505, Japan; asai@yamaguchi-u.ac.jp; 5 AI Systems Medicine Research and Training Center, Graduate School of Medicine and Yamaguchi University Hospital, Yamaguchi University, 1-1-1 Minami-Kogushi, Ube, Yamaguchi 755-8505, Japan; 6 LABEX, HEC-Lausanne, University of Lausanne, Quartier UNIL-Chamberonne, CH-1015 Lausanne, Switzerland; 7 Faculty of Law, Criminal Justice and Public Administration, University of Lausanne, Quartier UNIL-Chamberonne, CH-1015 Lausanne, Switzerland

**Keywords:** executive control, methylphenidate, ADHD subtype, attentional network task, conflict effect, executive control, dimensional analysis, ex-Gaussian parameters

## Abstract

Patients affected by Attention-Deficit/Hyperactivity Disorder (ADHD) are characterized by impaired executive functioning and/or attention deficits. Our study aim is to determine whether the outcomes measured by the Attention Network Task (ANT), i.e., the reaction times (RTs) to specific target and cue conditions and alerting, orienting, and conflict (or executive control) effects are affected by cognitive training with a Dual *n*-back task. We considered three groups of young adult participants: ADHD patients without medication (ADHD), ADHD with medication (MADHD), and age/education-matched controls. Working memory training consisted of a daily practice of 20 blocks of Dual *n*-back task (approximately 30 min per day) for 20 days within one month. Participants of each group were randomly assigned into two subgroups, the first one with an adaptive mode of difficulty (*adaptive* training), while the second was blocked at the level 1 during the whole training phase (1-back task, *baseline* training). Alerting and orienting effects were not modified by working memory training. The dimensional analysis showed that after *baseline* training, the lesser the severity of the hyperactive-impulsive symptoms, the larger the improvement of reaction times on trials with high executive control/conflict demand (i.e., what is called *Conflict Effect*), irrespective of the participants’ group. In the categorical analysis, we observed the improvement in such *Conflict Effect* after the *adaptive* training in adult ADHD patients irrespective of their medication, but not in controls. The ex-Gaussian analysis of RT and RT variability showed that the improvement in the *Conflict Effect* correlated with a decrease in the proportion of extreme slow responses. The Dual *n*-back task in the *adaptive* mode offers as a promising candidate for a cognitive remediation of adult ADHD patients without pharmaceutical medication.

## 1. Introduction

Attention-Deficit/Hyperactivity Disorder (ADHD) is a behavioral condition caused by a complex interplay between genetic and environmental risk factors affecting brain networks and leading to a broad range of impairments that interfere with functioning and development [1,2,3,4,5]. Although ADHD was initially considered to be a childhood-onset condition with limited effect on the adult, the symptoms associated with ADHD are increasingly observed in adults [6,7,8,9]. Higher-level executive functioning and emotional control deficits are more diverse in adult than in child ADHD patients [10,11]. The current mainstay of treatment of ADHD is medication: stimulant drugs such as methylphenidate and amphetamine [12] or non-stimulant drugs such as atomoxetine [13]. Several studies have reported that stimulants induced significant improvements in core symptoms of ADHD, in daily functioning and in executive impairments [14]. The stimulants have poor adverse effect profiles and a multitude of drug interactions [15] provoking non-serious adverse events as decreased appetite, insomnia, and sleep disturbances whose etiology is still complex and unclear [16]. Therapeutic approaches avoiding drug medication attract patients who resist taking stimulants and who want to avoid the risk of side effects of drugs. In this context, cognitive training, such as working memory (WM) training, gained interest as an alternative treatment of attentional and neuropsychological deficits in ADHD patients [17,18,19,20,21].

The Attention Network Task (ANT) [22] is a computer-based task that was developed to evaluate the function of attention, which can be broken down into the three components according to the attention network theory [23,24]. These components are meant to be functionally independent and corresponding to anatomically segregated networks, each one with different yet interrelated functions of selective attention called alerting, orienting and executive attention (or conflict) network [25,26,27]. The analysis of the reaction times (RTs) in the ANT provides an estimate of the preservation of each component of the attention network. Adult ADHD patients exhibited higher intra-individual variance and longer RTs than controls, in nearly all attentional tasks [28]. However, several arguments based on typical RT distributions to neuropsychological tasks in child ADHD pointed out that characterization of clinical heterogeneity should be based rather on the analysis of parameters (i.e., *mu*, *sigma*, and *tau*) derived from the ex-Gaussian distributional model of RTs [29,30,31,32]. A meta-analysis investigating the RT variability in controls and ADHD children, adolescents, and adults [33] concluded that the variability in the task performance of ADHD was primarily due to a set of abnormally slow responses, rather than ubiquitous variability across all trials in the task.

In contrast to increased mean RT, the distributional parameter *mu* did not document a significant slowing in adult ADHD and a significant correlation was reported between *tau* and the number of omission errors in a Go/NoGo task bringing evidence for attentional dysfunction [34]. However, there is yet no evidence of how a cognitive training of adult ADHD patients may affect the attention network. The alerting network is related to arousal and vigilance and is meant for achieving and maintaining a state of being very sensitive to incoming stimuli combined with a readiness to react. The orienting network is defined as selecting information from sensory input and shifting attention, i.e., disengaging and re-engaging attention. The conflict network comprises mechanisms for monitoring and resolving conflict among thoughts, feelings, and responses. Children with ADHD showed acceptable criterion validity for incorrect responses, omissions, and conflict score during ANT, despite high RT variability [35,36]. In child ADHD patients, the alerting scores on the ANT after WM training with an *n*-back task (NBT) were found to be a good predictor on math achievement [37].

The NBT is a popular experimental paradigm for WM training [38], in which subjects are asked to monitor two sources of information simultaneously (i.e., the specification and the location of a stimulus), with increasing attentional and working memory load, and decide if the currently presented stimulus is the same as the one presented in *N* trials previously [39,40]. The Dual *n*-back task (DNBT) is a version of the task eliciting divided attention because dual, one auditory and one visual, unrelated stimuli appear simultaneously and subjects are asked to monitor both stimuli independently [41]. DNBT received notable attention for its potential to improve WM and other aspects of cognitive performance [42,43]. The present study is the first one, to our knowledge, aimed at determining whether, in ADHD, the scores for the three ANT components measured by RTs and corresponding ex-Gaussian distributional model are selectively affected by WM training with a DNBT. We tested age-matched controls, medicated, and unmedicated young ADHD adults. In addition to group comparisons, we considered the dimensional analysis [44,45] to test whether improvement through training is associated with ADHD severity [46,47] on a continuum rated by Conners’ Adult ADHD Rating Scales-Self Report subscales. We found evidence that one-month training with DNBT has an impact on the executive control of any tested group, measured by the conflict network effect in ANT.

## 2. Materials and Methods

### 2.1. Participants

We recruited 114 (64 males and 50 females) young adults between 18 and 30 years old in the three groups of study, ADHD medicated with methylphenidate hydrochloride prescription ($N_{MADHD}=42$; 30 of these patients were diagnosed as a combined (mixed) subtype of ADHD (ADHD-C), 10 as predominantly inattentive subtype (ADHD-I), and 2 patients without any precise information provided by the psychiatrist regarding the subtype of the ADHD condition), ADHD without medication ($N_{ADHD}=34$; 18 of which were diagnosed as ADHD-C patients, 13 as ADHD-I, 1 as predominantly hyperactive/impulsive subtype (ADHD-HI), and 2 patients without any precise information regarding the subtype) and controls ($N_{CTL}=38$). All ADHD patients (N=76) were clinically referred by the Psychiatric Department of the University Hospital of Lausanne or at a psychiatrist’s practice in collaboration with the University Hospital after an initial screening appointment to ensure that they were fulfilling the criteria defined by the DSM-IV-TR for inattentive, hyperactive/impulsive, or mixed subtypes [48,49]. Under the supervision of a trained clinical psychologist, all participants underwent the Mini-International Neuropsychiatric Interview [50], a short structured diagnostic interview assessing psychiatric diseases, in order to exclude from this study those with ADHD comorbidity. We excluded from this study any individual referred taking mood and anxiety stabilizers, anti-depressants, any dopamine receptor-blocking drug and non-stimulant medications for ADHD.

The assignment of a participant to the ‘medicated ADHD group’ (MADHD) or to the ‘ADHD group without medication’ (ADHD) was decided by the psychiatrist in charge of the patients, on the exclusive basis of the patients’ therapeutic treatment. This assignment was ‘double-blind’, in the sense that the experimenters did not know which patient belonged to either ADHD group until the end of the protocol and the psychiatrist did not know which level of working memory training is assigned to a patient. Participants of the ADHD group with medication were required to stop medication 24 h prior to testing [51,52,53]. All participants underwent a short structured diagnostic interview to assess their psychiatric status. Control subjects were age/education-matched volunteer healthy controls screened prior to the experimental session to ensure that they would not report any neuropsychiatric diseases or any other exclusion criteria and none were taking any psychoactive medications. The study was carried out in accordance with the latest version of the Declaration of Helsinki [54] and approved by the mandatory Ethics Committees requested by Swiss Federal Authorities, following the constitutional article (art. 118b Cst) of 8 March 2010 and the Federal Act involving Human Beings on 30 September 2011 (revised 1 January 2014). All participants had normal or corrected-to-normal visions, none reported a history of sustained head injury. All participants were requested to fill out French versions of the adult ADHD Self-Report Scale (ASRS) and the Conners’ Adult ADHD Rating Scales-Self Report (Screening Version, CAARS-S:SV) [55,56,57] two weeks prior to the beginning of the protocol. All participants received monetary compensation following the scale approved by the mandatory Ethics Committees requested by Swiss Federal Authorities.

### 2.2. Experimental Protocol

The experimental procedure of this study included 3 parts. The first part was a *before-training* session of the ANT in the experimental laboratory (Labex, HEC-UNIL). The second part was a *WM training* period lasting one month with the Dual *n*-back task at home. The third part was an *after-training* session of the ANT at the same laboratory as the pre-test.

#### 2.2.1. Attention Network Task (ANT)

**Figure 1:**
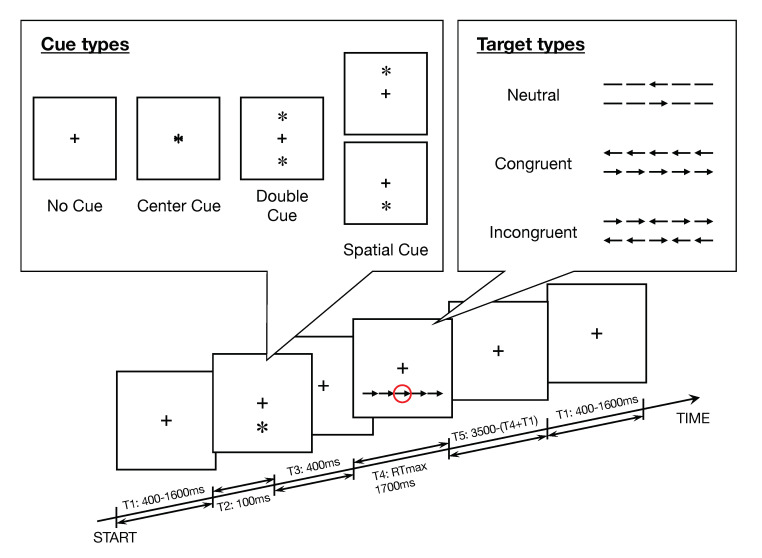
Experimental procedure of the Attention Network Task (ANT). The steps in a trial are summarized as follows: (T1) The participant is requested to fix a cross at the center of the screen for a random interval in the range 400–1600 ms. (T2) A cue appears for 100 ms, in the upper window, the four cue conditions. (T3) The cue disappears. (T4) A target stimulus with flankers appears, marked in the red circle in this figure; the related panel shows the six stimuli used in the present experiment. The participant is requested to select as quickly as possible the button corresponding to the direction of the target stimulus. In this example, the correct choice is to press the right button of the computer mouse. (T5) A final interval with participant’s gaze focused on the central cross is set until the next trial is started.

This task was originally reproduced from the original ANT [22]. Each trial had a fixed duration of 3500 ms and was formed by 5 successive intervals, as described in Figure 1. At the beginning of the trial, the participants were instructed to maintain their gaze on a black fixation cross at the center of a 19-inch computer screen with white background at a viewing distance of about 70 cm. After a uniformly distributed random interval lasting from 400 to 1600 ms, a cue, represented by an asterisk having the same size of the cross, appeared for 100 ms. We considered four cue types which were randomly distributed: *No Cue* (NC), *Center Cue* (CT, a cue superimposed to the cross at the center of the screen), *Double Cue* (DB, a cue appearing both above and below the cross), and *Spatial Cue* (SP_above or SP_below, a cue appearing either above or below the cross). The participants maintained their gaze on the black fixation cross during 400 ms, after the disappearance of the cue. Then, the participants were requested to identify a target (i.e., a target arrow) and determine its direction (left or right) as quickly as possible by pressing the corresponding button (left or right) of a computer mouse. The time taken to respond was recorded as reaction time (RT). Finally, the participants maintained their gaze on the black fixation cross during the remaining time till the end of the trial.

The target arrow (i.e., the target) was surrounded by flankers on both sides by 2 items, such that the target was located at the center of a line of 5 items. (Figure 1). Flankers presented above or under a fixation cross and corresponding to arrows pointing to the same direction of the target were labeled *Congruent* (CON), *Incongruent* (INC, if pointing to the opposite direction), or *Neutral* (NTL, simple lines). Errors (trials with an incorrect answer) and no responses (trials without response) were also recorded for each participant *i*, in order to calculate the *accuracy rate*. For each participant *i* we computed the *accuracy rate* ${\mathrm{AR}}_{i}=N_{(\mathrm{correct}\;\mathrm{trials})i}/N_{{\left(\mathrm{total}\;\mathrm{number}\;\mathrm{of}\;\mathrm{trials}\right)}_{i}}$. The combinations of 5 cue types (*No Cue, Center Cue, Double Cue, Spatial Cue_below, Spatial Cue_above*) × 3 target types ( *Neutral, Congruent, Incongruent*) × 2 directions ( *Left, Right*) defined 30 primary patterns of trials. After 1 practice block with 24 trials pseudorandomly selected ( [*No Cue, Center Cue, Double Cue, Spatial Cue_below, Spatial Cue_above*] × [*Neutral, Congruent, Incongruent*] × [*Left, Right*]), the participants performed 3 experimental blocks, each one including 96 trials, that means 288 trials overall for each session and for each participant. For data analysis, all trials with *Spatial Cue_below* and *Spatial Cue_above* were pooled together and all trials with targets oriented to the left or to right side were pooled together. Hence, the task included 12 final patterns, i.e.,  4 cue types (*No Cue, Center Cue, Double Cue, Spatial Cue*) × 3 target types (*Neutral, Congruent, Incongruent*).

#### 2.2.2. Computation of the Attention Network Effects

The effectiveness of the attention networks can be estimated from the RTs measured for the cue and target conditions [22]. The distribution of the RTs are not normally distributed but skewed towards long RTs (this aspect will be treated separately in ex-Gaussian analysis). Hence, the median values ($\tilde{x}$) of the RTs were chosen instead of the means for the calculation of each effect, as follows: *Alerting Effect* (AE) $={\tilde{RT}}_{No\;Cue}-{\tilde{RT}}_{Double\;Cue}$, *Orienting Effect* (OE) $={\tilde{RT}}_{Center\;Cue}-{\tilde{RT}}_{Spatial\;Cue}$ and *Conflict Effect* (CE) $={\tilde{RT}}_{Incongruent}-{\tilde{RT}}_{Congruent}$. The higher the *Alerting Effect* and *Orienting Effect* scores, the more efficient the networks are. For *Conflict Effect*, the lower the score, the more efficient its network. Notice that the median RT for each one of the 12 final patterns is hereafter referred simply as the RT.

#### 2.2.3. WM Training: Dual *n*-Back Task

**Figure 2:**
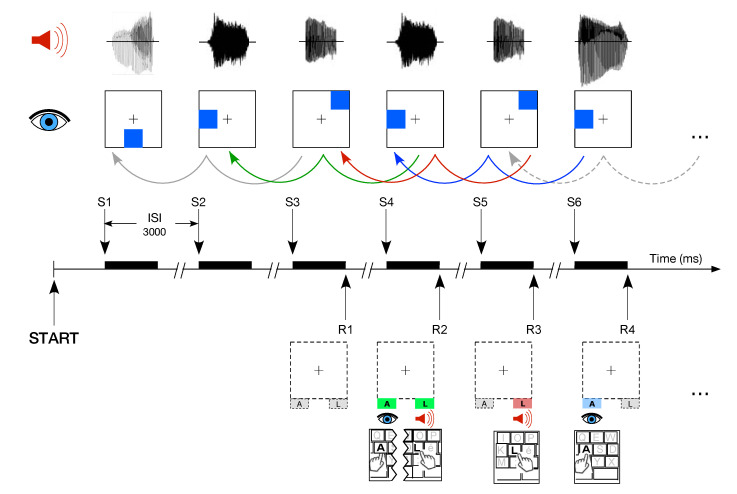
Example of level n=2 of the Dual *n*-back task. The task consisted of 20 blocks of at least 20 trials. Each trial was composed of an auditory and visual stimulus presented simultaneously. Participants were asked to detect and to press a key if any of the current stimuli corresponded to the one presented in the previous trial. They had to press the ‘A’ keyboard letter to report the correspondence with a visual target while the auditory target required the pressing of the ‘L’ key. Modified from [21].

The task consisted of two variants of the DNBT described in detail elsewhere [20,21,41]. The task is illustrated in Figure 2 and briefly summarized as follows. Each trial was composed of an auditory and visual stimulus presented simultaneously during 500 ms. The participants were asked to memorize the dual modality cues and to detect, by pressing a key, if any of the current stimuli correspond to the one presented in the previous trial (for 1-back). They had to press the ‘A’ keyboard letter to report the correspondence with a visual target, and to press the ‘L’ keyboard letter for matching the auditory target. The level of difficulty of the task is referred to as *n*-back. Then, if participants were required to detect a match with the previous trial, the mode is referred to as 1-back, called also the *baseline training*. In the *adaptive training*, the difficulty *n* of the task was adjusted as a function of the performance. The whole task consisted of 20 blocks of 20 + *n* trials with the same level of difficulty. An increase by 1 in the level of difficulty in the next block was triggered by a performance of less than 3 mistakes in each modality. With levels of difficulty higher than 1, a decrease by 1 in the level was triggered by 5 or more errors accumulated in any modality. In other cases, the level remained unchanged. The total duration of the working memory task was approximately half an hour.

During the *before-training* session, all participants performed one session of *adaptive* DNBT. Participants of each group were randomly assigned to either *baseline* or *adaptive* training mode for the one-month WM training period at home. It is very important to note that the random assignment to either group of training level was done in ‘blind’ mode to the experimenter, who knew only about the recruitment of controls vs. patients. This fact is very important for the comparison of MADHD and ADHD groups. This mode of assignment explains why the structure of ADHD and MADHD groups is not balanced with respect to the diagnosed subtype of ADHD (Table 1). Participants were asked to perform each weekday one session of DNBT via a secured web page, which allowed us to monitor whether the training sessions were completed correctly. In case of problems, the participants were advised to complete the incomplete sessions during the weekends. Those who did not successfully complete at least 18 sessions were excluded from this study. At the *after-training* session, all participants performed again one *adaptive* DNBT.

### 2.3. Statistical Analysis

R 4.0.0 statistical software was used in all the analyses [58], with packages outliers [59], WRS2 [60], robust [61] effectsize [62], coin [63], lmtest [64], and report [62]. The non-parametric test for paired groups (Wilcoxon signed-rank test) was applied to test the effect of training before and after the working memory training (WMT) at home with the dual *n*-back task. The Mann–Whitney test was used for the unpaired two-groups comparisons. Factorial analysis was performed to test interactions among groups (3 ‘group’ factors: *CTL, MADHD, ADHD*), between sessions (2 ‘session’ factors: *before-training*, *after-training*), and between WM training modes (2 ‘level’ factors: *baseline*, *adaptive*). We assume the null hypothesis of homoscedasticity in the data samples and test it with the studentized Breusch–Pagan test. The standard ANOVA can be used for factorial analysis if the value of the test statistics is not significant and partial omega squared ($\omega_{p}^{2}$) is used to test the effect size. The magnitude of effect sizes is labeled as follows: *statistically insignificant* as “+si”: $\omega_{p}^{2}\;<\;0.01$; *small* as ”+s”: $0.01\leq \omega_{p}^{2}\;<\;0.06$; *medium* as “+m”: $0.06\leq \omega_{p}^{2}\;<\;0.14$; *large* as “+L”: $0.14\leq \omega_{p}^{2}$. Notice that if the value of the observed *F* is less than one $\omega_{p}^{2}$ will be negative. In addition to being insignificant, a value of *F* less than one is a sign of inconsistency in the statistics. For the Student’s *t*-test, the effect size is assessed by Cohen’s *d* with magnitudes: *statistically insignificant* as “+si”: d < 0.2; *small* as ”+s”: 0.2≤d < 0.5; *medium* as “+m”: 0.5≤d < 0.8; *large* as “+L”: 0.8≤d.

In case homoscedasticity is rejected, we apply robust versions of ANOVA, i.e., heteroscedastic one-way ANOVA for medians and two- and three-way ANOVAs with trimmed means at level 0.2 and effect size assessed by $\hat{\xi }$ with magnitudes: *statistically insignificant* as “+si”: $\hat{\xi }\;<\;0.1$; *small* as ”+s”: $0.1\leq \hat{\xi }\;<\;0.3$; *medium* as “+m”: $0.3\leq \hat{\xi }\;<\;0.5$; *large* as “+L”: $0.5\leq \hat{\xi }$. For non-parametric Wilcoxon Signed-Rank and Mann–Whitney tests, the effect size is assessed by *r* with magnitudes: *statistically insignificant* as “+si”: r < 0.1; *small* as ”+s”: 0.1≤r < 0.3; *medium* as “+m”: 0.3≤r < 0.5; *large* as “+L”: 0.5≤r. Notice that there are no available tests for heteroscedastic ANOVA with unbalanced repeated measurements. The computation of the ex-Gaussian parameters (*mu*, *sigma*, and *tau*) were computed with the mexgauss function package retimes [65]. In general, the grouped values are reported as (median, average ±, and SEM).

## 3. Results

### 3.1. Unsupervised Exclusion of Outliers

We applied an unsupervised procedure aimed at excluding participants characterized by an outlier performance. In order to do so, we conducted a two steps procedure and we applied the scores function of the package outliers of the R statistical software throughout the study. The first step was aimed at excluding from the analysis the participants characterized by an outlying performance either before or after the WM training. For each participant *i* we computed the *accuracy rate* ${\mathrm{AR}}_{i}$. We tested the normality of the distributions of ${\mathrm{AR}}_{i}$ using the Shapiro–Wilk test for each group, before and after training. In all groups, the distribution of AR was not following a normal distribution. Hence, we applied the scores function with robust estimation of the differences between each value and median, divided by median absolute deviation (“mad”). According to the size of our samples the *Z* score of the outliers was bounded by the value $\left(n-1\right)/\sqrt{n}$ [66]. The outcome of this procedure was the removal of 5 participants (2 controls, 1 MADHD, and 2 ADHD).

In order to reduce the impact of outliers in the skewed distribution of RTs we applied a logarithmic transformation of RTs for all trials [67,68]. The second step of the procedure consisted in detecting the outlier trials using the log-transformed RTs measured for each one of the 30 primary trial patterns (see Section 2.2.1) using the scores function based on the median absolute deviation. On average we observed 18.7/288, 17.6/288, and 17.8/288 outlier trials for each participant belonging to controls, MADHD and ADHD, respectively. After removal of all outlier trials for all participants, the median RT was computed for each primary trial pattern. Then, within each group and for each such primary trial pattern, the participant outliers were detected after applying the logarithmic transformation to the series of median RTs. Any participant with more than 1 outlier median RT in the group series of any primary trial pattern was checked out as an outlier participant. The outcome of this second procedure was the removal of additional 4 participants (1 control, 1 MADHD, and 2 ADHD). At the end of both procedures for removal of outlier participants, the overall sample of the remaining 104 participants included 27,719 trials before training and 27,460 trials after training. The accuracy rates of all participants, irrespective of their groups and subgroups, were above 95% (Table 1) and all values were homoscedastic (BP=3.82, df=5, p=0.58 and BP=3.47, df=5, p=0.63, before- and after-training, respectively). The outlier trials represented 6.40% and 6.98% of the total number of valid trials before and after WMT, respectively.

### 3.2. Clinical Assessment Scales and Subscales

A quantitative assessment of clinical symptoms fulfilling the criteria defined by the DSM-IV-TR for inattentive, hyperactive/impulsive or mixed ADHD subtypes [48] was performed using the ADHD Self-Report Scale (ASRS) [56] and Conners’ Adult ADHD Rating Scales-Self Report (Screening Version, CAARS-S:SV) [55,57]. After removal of the outliers the final sample sizes of patients were $N_{MADHD}=40$ including 28 ADHD-C, 10 ADHD-I, and 2 unknown subtype and $N_{ADHD}=30$ including 16 ADHD-C, 12 ADHD-I, 1 ADHD-HI, and 1 unknown subtype. We consider a model where ASRS [56] and the normalized T-score of CAARS, referred to as the ‘ADHD Index’ [55,57], depend on three factors: *patients’ group* × *ADHD subtype* × *level*, where *level* refers to the level of training assigned to each participant before WMT with the Dual *n*-back task.

For ASRS, the null hypothesis for homoscedasticity and homogeneity of variances were accepted (BP=6.12, df=7, p=0.53 and Levene’s test F(7,58)=0.80, p=0.59), then standard ANOVA could be applied. Two main effects were significant, i.e., factor *ADHD subtype* (F(1,58)=13.65, p < 0.001, effect size $\omega_{p}^{2}=0.16$ +L) and factor *group* (F(1,58)=8.25, p < 0.01, $\omega_{p}^{2}=0.10$ +m). In both groups of patients, the ASRS score of ADHD-C patients was significantly higher than the score of the predominantly inattentive subtype (${\mathrm{ASRS}}_{MADHD}\left(ADHD-C\right)=69.5\pm 2.2$ vs. ${\mathrm{ASRS}}_{MADHD}\left(ADHD-I\right)=57.2\pm 2.5$ and ${\mathrm{ASRS}}_{ADHD}\left(ADHD-C\right)=62.2\pm 2.3$ vs. ${\mathrm{ASRS}}_{ADHD}\left(ADHD-I\right)=54.0\pm 2.9$). It is important to notice that the interaction between *group* and *ADHD subtype* is not significant (F(1,58)=0.25, p=0.621, $\omega_{p}^{2}=-0.01$ +si). Notice that this last *F* value is less than one and the lack of significant interaction mean that the effect of patients’ group is independent from the ADHD subtype. Table 1 includes also the controls and it shows the median, mean, and SEM of the clinical assessment scales and subscales for subgroups *baseline* and *adaptive*, as defined by the level of training during one month with the Dual *n*-back task, for each group of participants. The two-way ANOVA showed that the main effect of *group*, including also the group of controls, was very significant (F(2,99)=25.65, p < 0.001, $\omega_{p}^{2}=0.32$ +L). We observed neither a main effect of the level of training assigned to the subgroups, nor an interaction between the factors, then we considered the two groups of patients irrespective of the training level. The ASRS scores were ${\mathrm{ASRS}}_{CTL}$ (47.0, 47.0 ± 1.9), ${\mathrm{ASRS}}_{MADHD}$ (65.5, 65.7 ± 1.9), and ${\mathrm{ASRS}}_{ADHD}$ (58.5, 57.9 ± 1.9). Tukey post-hoc multiple comparisons showed that the ASRS scores of controls were different from patients’ groups (t(73)=6.98, p < 0.001, effect size d=1.6 +L and t(63)=4.09, p < 0.001, d=1.0+L for MADHD and ADHD, respectively). Notice that ASRS was also different from each other patients’ group (t(67)=2.89, p < 0.01, d=0.69 +m).

One participant belonging to the MADHD group (subgroup *baseline*) did not complete the CAARS questionnaire. For the DSM-IV Inattentive Symptoms Subscale (CAARS:A) the values were homoscedastic (BP=3.61, df=2, p=0.16). If we test the model with two factors *group*×*level of training* we found a strong effect of *group* (F(2,98)=51.85, p < 0.001, $\omega_{p}^{2}=0.49$ +L) and a small effect of the assigned *level of training* (F(1,98)=5.35, p < 0.05, $\omega_{p}^{2}=0.04$ +s). The smallness of this effect was confirmed by the lack of significance in the difference of the DSM-IV Inattentive Symptoms between the subgroups assigned to *baseline* and *adaptive* levels of WMT irrespective of the group of participants (t(101)=1.67, p=0.10, d=0.33 +s). Tukey post-hoc multiple comparisons showed that controls were significantly different from MADHD and ADHD (t(58)=6.66, p < 0.001, d=1.7 +L) and t(67)=10.18, p < 0.001, d=2.4 +L, respectively), but the inattentive symptoms of patients’ groups were not different from each other group (t(52)=1.76, p=0.09, d=0.43 +s). A separate three-way ANOVA limited only to the ADHD patients diagnosed as ADHD-C and ADHD-I testing the model of CAARS:A with factors *group*×*level of training*×*ADHD subtype* showed no significant main effect (*group*:F(1,57)=3.22, p=0.08, $\omega_{p}^{2}=0.03$ +s; *ADHD subtype*:F(1,57)=0.44, p=0.51, $\omega_{p}^{2}=-0.009$ +si; *level*:F(1,57)=1.92, p=0.17, $\omega_{p}^{2}=0.01$ +s).

The values of the DSM-IV Hyperactive-Impulsive Symptoms Subscale (CAARS:B) were heteroscedastic (BP=22.15, df=5, p < 0.001) and we used a robust two-way ANOVA for factors *group*×*level*. Table 1 shows that the outcome of the comparisons of the DSM-IV Hyperactive-Impulsive Symptoms Subscale (CAARS:B) was similar to the Inattentive Symptoms Subscale, that is a large main effect of *group* (Q=47.3, p=0.001, effect size $\hat{\xi }=0.87$ +L) and a small effect of the assigned *level of training* (Q=6.4, p < 0.05, $\hat{\xi }=0.29$ +s). The differences between the CAARS:B scores of the subgroups were not significant for controls and medicated ADHD (U=106.5, Z=1.54, p=0.13, r=0.26 +s and U=141.5, Z=1.36, p=0.18, r=0.22 +s, respectively), and just below the threshold (5%) for ADHD (U=64.5, Z=1.98, p=0.0497, r=0.36 +m ). In a separate analysis limited to patients’ groups MADHD and ADHD, we observed a significant main effect of factor *ADHD subtype* (Q=60.8, p < 0.001, $\hat{\xi }=0.64$ +L). In both patients’ groups, the values of the DSM-IV Hyperactive-Impulsive Symptoms Subscale of ADHD-C patients were significantly higher than the values of ADHD-I (for MADHD: $\mathrm{CAARS}:B_{ADHD-C}=68.9\pm 2.7$ vs. $\mathrm{CAARS}:B_{ADHD-I}=42.1\pm 2.5$, Mann–Whitney test U=256, Z=4.14, p < 0.001, r=0.68 +L and for ADHD: $\mathrm{CAARS}:B_{ADHD-C}=67.1\pm 1.9$ vs. $\mathrm{CAARS}:B_{ADHD-I}=56.4\pm 3.2$; U=151, Z=2.57, p < 0.05, r=0.48 +m).

Standard ANOVA was used to analyze the model of the DSM-IV Total ADHD Symptoms Subscale (CAARS:C) as a function of factors *group*×*ADHD subtype*×*level of training* because the values were homoscedastic (BP=7.08, df=7, p=0.42 and variances were homogeneous F(7,57)=1.26, p < 0.001). The difference in the values of CAARS:C scores between MADHD and ADHD was not significant (t(61)=1.76, p=0.64, d=0.11 +si) but the main effect of *ADHD subtype* was significant (F(1,57)=20.18, p < 0.001, $\omega_{p}^{2}=0.23$ +L). The values of CAARS:C score of patients diagnosed with the combined subtype were larger than those diagnosed as predominantly inattentive (for MADHD: $\mathrm{CAARS}:C_{ADHD-C}=77.9\pm 2.1$ vs. $\mathrm{CAARS}:C_{ADHD-I}=60.5\pm 2.1$, t(27)=5.82, p < 0.001, d=1.9 +L) and for ADHD: $\mathrm{CAARS}:C_{ADHD-C}=74.7\pm 2.3$ vs. $\mathrm{CAARS}:C_{ADHD-I}=67.9\pm 3.7$; t(19)=1.56, p < 0.001, d=0.61 +m). The ANOVA extended to the controls showed a large main effect of *group* (F(2,98)=47.16, p < 0.001, $\omega_{p}^{2}=0.47$ +L). The main effect of the assignment to the *level* of training was significant (F(1,98)=8.86, p=0.004, $\omega_{p}^{2}=0.07$ +m) and the comparison between the *baseline* and *adaptive* subgroups, irrespective of the participant’s group was also significant t(102)=2.21, p < 0.05, d=0.43 +s). However, a more detailed analysis for patients’ groups showed no difference of CAARS:C score between subgroups assigned to *baseline* or *adaptive* protocol (for MADHD: t(35)=1.49, p=0.14, d=0.48 +s and for ADHD: t(26)=1.63, p=0.12, d=0.59 +m). It is within the control group, and not in any patients’ group, where the participants assigned to different WMT protocols showed a difference in Total ADHD symptoms (t(32)=2.21, p < 0.05, d=0.76 +m), which provoked the main effect in the factor *level*. Even with a small significance, any main effect associated with the assignment to the *level* of training should be considered carefully because it could suggest a potential bias in the outcome of the random assignments of the participants to either *baseline* or *adaptive* subgroups prior to training.

For the ‘ADHD Index’ (i.e., the normalized T-score of CAARS [55,57]), we tested again the null hypothesis for homoscedasticity and homogeneity of variances against a model with factors *ADHD subtype*, in addition to factors *group* and *level*. The null hypothesis was accepted for this model (BP=7.43, df=7, p=0.39 and Levene’s test F(7,57)=0.71, p=0.67), then standard ANOVA could be applied. In patients’ groups comparison, the main effect of *group* was significant (F(1,57)=6.39, p < 0.05, $\omega_{p}^{2}=0.08$ +m) but the main effect of the *ADHD subtype* was not and no significant interaction was found between factors in the ANOVA. It is noteworthy that in ADHD patients without medication (and only in that group), the ADHD index of the patients diagnosed with a combined ADHD subtype (60.5, 61.0±1.9) was significantly (t(25)=2.46, p < 0.05, d=0.93 +L) larger than the ADHD index of those diagnosed with a predominantly inattentive ADHD subtype (54.0, 54.2±2.0). The two-way ANOVA, also including the group of controls (Table 1), shows a significant main effect of the *group* factor (F(2,98)=29.28, p < 0.001, $\omega_{p}^{2}=0.35$ +L) with differences between controls and patients (with MADHD: t(69)=7.30, p < 0.001, d=1.7 +L and with ADHD: t(61)=5.03, p < 0.001, d=1.3 +L) and between MADHD and ADHD t(67)=2.70, p=0.01, d=0.6 +m) in a way somehow similar to what we observed with ASRS. On the contrary, the main effect of the *level* factor was not significant (F(1,98)=3.02, p=0.09, $\omega_{p}^{2}=0.02$ +s), such that we may consider that the subgroups defined by the random assignment to either *baseline* and *adaptive* memory training are not clinically biased with respect to the ’ADHD Index’.

### 3.3. Dimensional Analysis of Reaction Time (RT)

**Figure 3:**
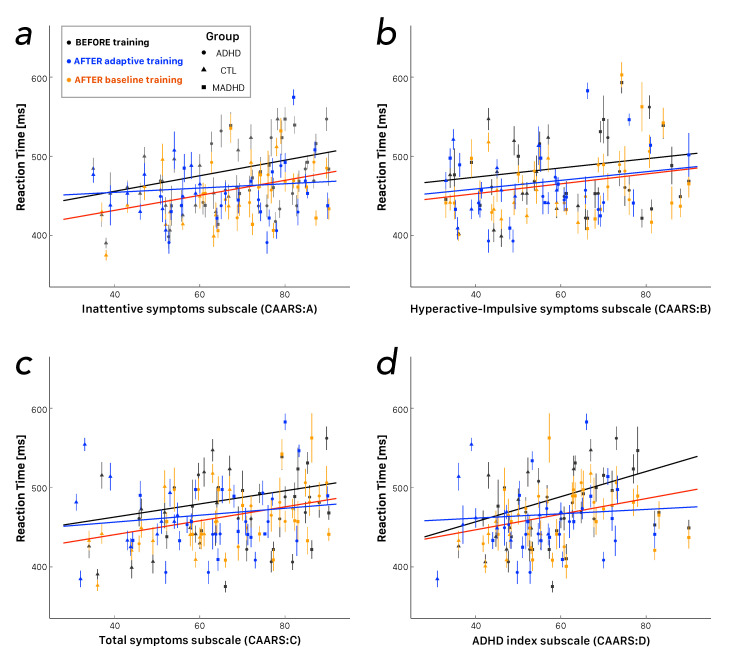
Dimensional analysis of reaction times as a function of the severity of ADHD symptoms measured by Conners’ Adult ADHD Rating Scales-Self Report (Screening Version, CAARS-S:SV). (**a**) Scatterplot as a function of CAARS:A (*DSM-IV Inattentive Symptoms Subscale*). (**b**) Scatterplot as a function of CAARS:B (*DSM-IV Hyperactive-Impulsive Symptoms Subscale*). (**c**) Scatterplot as a function of CAARS:C (*DSM-IV Total ADHD Symptoms Subscale*). (**d**) Scatterplot as a function of CAARS:D (*‘ADHD Index’, the normalized T-score of CAARS*). Each point shows the median and the median absolute deviation for one participant. Participants’ groups are identified by distinct shapes, i.e., triangles for controls, circles and squares for non-medicated and medicated ADHD patients, respectively. Data points before training (all subgroups merged together) are plotted in black. Data points after training are plotted in red for the *baseline* level (fixed at 1-back) and in blue for the *adaptive* level of the Dual *n*-back. Color lines refer to the robust regression of the corresponding data points. Notice that the slope for participants before training and after *baseline* training tended to be very similar. Notice also that the slopes tended to flatten after *adaptive* training, irrespective of the CAARS subscale.

The dimensional analysis assumes that a measured variable depends on the severity (i.e., intensity) of the symptoms rated on a continuous scale. In the current study, this assumption was that the reaction times should be tested against the four scores derived from the analysis of the CAARS questionnaire (i.e., CAARS:A, CAARS:B, CAARS:C, and CAARS:D), irrespective of the participants’ groups. Figure 3 shows the corresponding scatterplots and the regression lines between the reaction times and the CAARS subscales. The correlation before training (black lines) and after the *baseline* training (red lines) tended to be characterized by the same slopes. The intercepts after the *baseline* training were smaller, thus suggesting the training produced an increased speed in the reaction time, which was the same for light or severe values of the symptoms, measured by CAARS subscales. It is interesting to note that the *adaptive* training tended to show lines becoming flat, thus suggesting a decrease in the reaction time, which tended to be independent of the severity of the symptoms. The only exception is illustrated by the correlations with the CAARS:B (i.e., the *DSM-IV Hyperactive-Impulsive Symptoms Subscale*, Figure 3b) with an increased speed, which tended to be the same for both training modes, irrespective of the severity of the symptoms (i.e., parallel blue and red lines). Each pattern of the ANT was tested several times for each participant, such that we should be able to use repeated measurements in statistical tests. The design of the experiment was balanced, but several trials were discarded due to the fact that either the participants did not answer within the time limit or the trial fell among the outliers. Hence, the outcome was unbalanced with respect to an ideal repeated measurements design for the number of trials in any combination of ANT patterns and training protocol.

We tested the null hypothesis of homoskedasticity with a studentized Breusch–Pagan test for RTs as a function of each CAARS score before and after training. In all conditions, we found that the RTs were heteroscedastic, such that we should apply robust ANOVA with repeated measures. The main effects were significant for all scores, i.e., CAARS:A (F(1,206)=11.04, p < 0.01, $\omega_{p}^{2}=0.05$ +s), CAARS:B (F(1,206)=7.72, p < 0.01, $\omega_{p}^{2}=0.03$ +s), CAARS:C (F(1,206)=11.13, p < 0.01, $\omega_{p}^{2}=0.05$ +s), and CAARS:D (F(1,206)=13.10, p < 0.001, $\omega_{p}^{2}=0.06$ +m). Then, we analyzed the correlations by means of Spearman’s rank correlation ρ and the corresponding robust linear regression between RTs and the CAARS scores (Table 2). We observed that the more severe the symptoms the longer the RT, except for most scores of the ADHD group without medication after *adaptive* training.

### 3.4. Categorical Analysis of Reaction Time (RT)

We consider at first the RTs before training to all task patterns for further assessment of a potential bias introduced by the initial assignment of a participant to one of the training protocol subgroups. Before training, the RTs of the participants assigned to the *baseline* mode showed only small differences among the groups (468.2±4.7, 488.1±4.6, and 484.9±4.9 ms for controls, MADHD, and ADHD, respectively) without any significant main effect of factor *group* by robust one-way ANOVA (Q=1.99, p=0.18, $\hat{\xi }=0.16$ +s). On the contrary, the main effect of factor *group* was significant among participants assigned to the *adaptive* subgroups before training (Q=10.75, p=0.001, $\hat{\xi }=0.26$ +s). In particular, the RTs of MADHD (511.3, 521.6 ± 6.0 ms) were significantly longer than controls (476.5, 486.5 ± 5.1 ms; Mann–Whitney test U=18,246.0, Z=3.74, p < 0.001, r=0.19 +s) and longer than ADHD (453.0, 473.3 ± 5.6 ms; U=13,229.5, Z=6.63, p < 0.001, r=0.26 +s). Any effect associated with the assignment of the participants to the *level* of *baseline* or *adaptive* protocol before WMT might reveal an initial bias in the composition of the groups. This is an important point to consider because of the limited size of our samples.

We have previously pointed out the fact that there is an unbalanced distribution of ADHD subtypes between MADHD and ADHD groups, in particular between predominantly inattentive type (ADHD-I) and combined (ADHD-C) subtypes (Table 1). For this reason we consider a model where the RTs of the patients depend on three factors: *patients’ group* × *ADHD subtype* × *training level*. The RTs were heteroscedastic (BP=32.747, df=7, p < 0.001) and variances were not homogeneous (Levene’s Test F(7,784)=3.88, p < 0.001), hence a three-way robust ANOVA was used. The only significant main effect of this analysis was due to factor *group* (Q=23.37, p < 0.001, effect size $\hat{\xi }=0.22$ +s). However, a barely significant triple interaction between *group* and *ADHD subtype* and *training level* (Q=4.68, p < 0.05, $\omega_{p}^{2}=0.004$ +si) calls for further insight. The assignment of the patients to the training level subgroups was done at random, at the very beginning of the experimental protocol. Before training, for MADHD patients assigned to the *baseline* subgroup, the overall (all ANT patterns pooled together) RTs of ADHD-C were shorter than the RTs of ADHD-I (484.0, 484.0 ± 5.4 ms vs. 496.2, 513.1 ± 9.8 ms; Mann–Whitney test, U=3519.0, Z=2.53, p < 0.05, r=0.16 +s). On the contrary, the RTs of ADHD patients without medication diagnosed as ADHD-C were longer than the RTs of ADHD-I (488.2, 499.9 ± 7.6 ms vs. 464.8, 469.9 ± 5.9 ms; U=5593.5, Z=2.56, p < 0.05, r=0.18 +s). In the *adaptive* subgroups of both ADHD and MADHD, no significant difference of RTs between ADHD-C and ADHD-I patients were observed before training. In view of these results, we may consider the interaction before training between factors *ADHD subtype* and *training level* as a spurious effect of the random sampling. For the patients’ groups, we analyzed the effect of factors *group* × *session* × *ADHD subtype* with all RTs to any task pattern pooled together. We found no main effect of factor *ADHD subtype* (Q=0.36, p=0.56, $\hat{\xi }=0.05$ +si), a significant main effect of factor *group* (Q=16.81, p < 0.001, $\hat{\xi }=0.14$ +s), a significant main effect of factor *session* (Q=27.14, p=0.001, $\hat{\xi }=0.21$ +s), and a significant interaction *group*×*session* (Q=6.20, p < 0.05, $\omega_{p}^{2}=0.003$ +si). Hence, due to the smallness of the subgroups divided according to the *ADHD subtype* and to the fact that this factor produced neither a main effect nor a significant interaction in the previous robust ANOVAs, the following analyses discarded the factor *ADHD subtype*.

**Figure 4:**
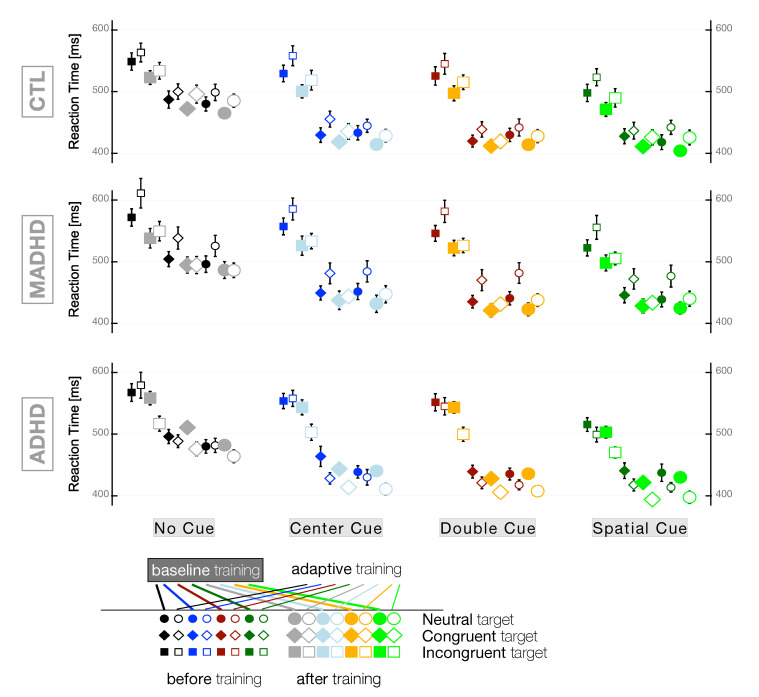
Reaction Times (means ± SEM) for all conditions: *Cue* × *Target* × *Group* × *Training level*. Participants’ groups: CTL: control subjects; MADHD: ADHD patients with medication; ADHD: ADHD patients without medication.

Figure 4 shows the RTs of all participants’ groups for any combination of the 12 final task patterns (*Target* × *Cue*) before and after training with *baseline* and *adaptive* levels. At first, we analyze the effect of factors *group* × *session* × *level* irrespective of any task patterns pooled together. The outcome of this robust ANOVA is a significant main effect of factor *group* (Q=31.80, p < 0.001, $\hat{\xi }=0.14$ +s), a significant main effect of factor *session* (Q=43.79, p < 0.001) $\hat{\xi }=0.20$ +s), and a significant *group*×*level* two-way interaction (Q=35.31, p=0.001, $\omega_{p}^{2}=0.02$ +s). Because of such interaction term, we analyzed separately the RTs for the two subgroups of training modes with two-way robust ANOVAs ($N_{observations}=1320$ and $N_{observations}=1200$, for *baseline* and *adaptive*, respectively). In both training modes we found a significant main effect for factor *group* (Q=22.34, p=0.001, $\hat{\xi }=0.22$ +s and Q=40.41, p=0.001, $\hat{\xi }=0.17$ +s, for *baseline* and *adaptive*, respectively) and a significant main effect for factor *session* (Q=12.35, p=0.001, $\hat{\xi }=0.15$ +s and Q=32.98, p=0.001, $\hat{\xi }=0.25$ +s, for *baseline* and *adaptive*, respectively).

In controls, the comparison of the RTs with their respective pre-training values by a Wilcoxon Signed-Rank test showed that both training modes produced faster reactions with a decreasing in RT of the same magnitude (after *baseline* training: −16.0, −18.6 ± 2.4 ms and V=4537.0, Z=6.80, p < 0.001, r=0.46 +m; after *adaptive* training: −16.0, −20.4 ± 3.0 ms and V=4085.5, Z=6.35, p < 0.001, r=0.45 +m). In controls, a robust one-way ANOVA to test the main effect for factor *session* in each training protocol showed a similar result, but to a lesser degree of significance (after *baseline* training: Q=11.74, p < 0.01, $\hat{\xi }=0.18$ +s; after *adaptive* training: Q=7.07, p=0.01, $\hat{\xi }=0.19$ +s). In medicated ADHD, both training modes produced faster reactions, but after one month of *adaptive* training (−39.5, −44.4 ± 4.5 ms and V=3242.0, Z=9.11, p < 0.001, r=0.61 +L) the effect was larger than after *baseline* training (−23.0, −18.9 ± 2.9 ms and V=6170.0, Z=7.51, p < 0.001, r=0.48 +m). The factorial analysis for MADHD was also in line with this observation (after *baseline* training: Q=10.44, p < 0.01, $\hat{\xi }=0.18$ +s; after *baseline* training: Q=15.40, p < 0.001, $\hat{\xi }=0.36$ +m). In ADHD without medication, the decrease in overall RTs after *adaptive* training was even stronger than the effect observed in MADHD (−23.5, −26.5 ± 2.9 ms; V=3242.0, Z=8.14, p < 0.001, r=0.64 +L). However, in ADHD without medication, one month training in *baseline* mode produced faster reactions below the threshold of significance (−7.5, −6.6 ± 3.7 ms; V=7029.5, Z=2.48, p=0.14, r=0.11 +s). The robust ANOVA showed that the main effect for factor *session* was neither significant for the *baseline* subgroup (Q=0.80, p=0.79, $\hat{\xi }=0.06$ +si) nor for the *adaptive* subgroup (Q=2.45, p=0.09, $\hat{\xi }=0.21$ +s). The discrepancy that we observed for the *adaptive* subgroup between the Wilcoxon Signed-Rank test and the robust one-way ANOVA for medians called to test the factor *session* also with the robust one-way ANOVA with trimmed means, which yielded a significant result after *adaptive* training F(1,196)=5.98, p < 0.05, $\hat{\xi }=0.21$ +s). This may be explained by the evaluation of significance in the algorithm of robust one-way ANOVA for medians, which is biased towards the safe side and tends to underestimate the level of significance, as mentioned by the authors of the method [60].

We can observe a general pattern of RTs as a function of the *cue* and *target* types irrespective of the group and training mode: The larger information in the cue (i.e., ‘spatial cue’) the shorter the RT, the more neutral the target the shorter the RT (Figure 4). The two-way robust ANOVA showed a significant main effect of factor *cue* (Q=358.475, p=0.001, $\hat{\xi }=0.39$ +m), a significant main effect of factor *target* (Q=1153.49, p=0.001, $\hat{\xi }=0.79$ +L), and a significant *cue*×*target* two-way interaction (Q=53.84, p < 0.001, $\omega_{p}^{2}=0.02$ +s). No significant difference was observed between RTs following congruent and neutral targets U=360,158.5, Z=0.74, p=0.46, r=0.02 +si). Because of this finding and for sake of simplicity, we skipped further analysis of the neutral targets and we focused on the differences between incongruent and congruent targets. In all groups and subgroups, we observed shorter RTs for congruent than incongruent targets (Table 3). The factorial *group*×*training*×*session* analysis showed main effects for factors *group* (Q=19.10, p < 0.001, $\hat{\xi }=0.13$ +s) and *session* (Q=33.67, p < 0.001, $\hat{\xi }=0.22$ +s) and a significant two-way interactions *group*×*training* (Q=22.05, p=0.001, $\omega_{p}^{2}=0.02$ +s). Table 3 shows that the source of the interaction was mainly due to what happened in the ADHD group of participants.

Non-medicated ADHD patients showed a significant difference in RTs after *adaptive* training for both incongruent and congruent targets (V=25.0, p < 0.001, r=0.83 +L and V=281.0, p < 0.001, r=0.50 +m, respectively). It is worth noting that no significant difference in RTs was observed in ADHD after *baseline* training (V=730.5, p=0.12, r=0.19 +s) and V=702.5, p=0.34, r=0.12 +s, for incongruent and congruent targets, respectively). For controls and MADHD, the decrease in RTs was significant after either kind of training mode irrespective of the target, with a similar effect of the WMT protocols in controls (Q=3.94, p=0.027, $\hat{\xi }=0.18$ +s and Q=3.89, p=0.025, $\hat{\xi }=0.20$ +s for *baseline* and *adaptive*, respectively) and with a stronger effect after *adaptive* training in medicated ADHD (Q=8.57, p=0.004, $\hat{\xi }=0.36$ +m vs. Q=3.21, p=0.037, $\hat{\xi }=0.18$ +s after *baseline* training).

A comparison of cue types showed that RTs during the ANT depended on the amount of information contained in the cue. In the absence of information (‘*No Cue*’), RTs were longer and in the presence of unambiguous comprehensive information (‘*Spatial Cue*’), RTs were shorter. For sake of simplicity, we focus further analyses on these extreme cue conditions and skip the data obtained for ‘*Center Cue*’ and ‘*Double Cue*’ (Table 4). For both *No Cue* and *Spatial Cue* conditions, the factorial *group* × *training* × *session* analysis showed main effects of *group* (Q=7.17, p < 0.05, $\hat{\xi }=0.14$ +s and Q=15.32, p < 0.001, $\hat{\xi }=0.18$ +s, for *No Cue* and *Spatial Cue*, respectively) and of *session* (Q=13.31, p < 0.001, $\hat{\xi }=0.20$ +s and Q=15.43, p < 0.001, $\hat{\xi }=0.23$ +s, for *No Cue* and *Spatial Cue*, respectively). In addition, we observed significant two-way interactions *group* × *training* (Q=8.24, p < 0.05, $\omega_{p}^{2}=0.01$ +s and Q=14.96, p < 0.001, $\omega_{p}^{2}=0.02$ +s, for *No Cue* and *Spatial Cue*, respectively). This interaction effect was due to ADHD (i.e., the patients without medication), which is the only group characterized by a very strong lack of significance of *baseline* training on RTs in *No Cue* (V=501.0, Z=0.03, p=0.95, r=0.00 +si) and in *Spatial Cue* (V=398.0, Z=1.84, p=0.18, r=0.19 +s) conditions. In any other combination of group and training mode, the RTs were significantly shorter after one month of WMT and stronger effect after *adaptive* training in patients’ groups.

### 3.5. Attention Network Effects

In a three-way ANOVA similar to the previous analyses, we tested whether the factor *ADHD subtype* affected the values of each attention network effect in the MADHD and ADHD groups and corresponding subgroups of patients randomly assigned to the *baseline* and *adaptive* levels of WMT. No heteroscedastic and no inequality of variances was observed for all network effects, thus allowing the standard parametric ANOVA to be applied for the statistical analysis. No main effect of factor *ADHD subtype* was found in the orienting and conflict networks (F(1,58)=3.04, p=0.09, $\omega_{p}^{2}=0.03\;+s$ and (F(1,58)=0.24, p=0.63, $\omega_{p}^{2}=-0.01\;+si$, respectively). On the contrary, the analysis of the *Alerting Effect*, showed a significant main effect of *ADHD subtype* (F(1,58)=8.56, p < 0.01, $\omega_{p}^{2}=0.10$ +m). In both patients’ groups, the value of *Alerting Effect* of diagnosed ADHD-C (MADHD: 56.2±4.7; ADHD: 56.6±5.3) was higher than the value of ADHD-I (MADHD: 34.8±7.7 ADHD: 42.3±5.6), but only in the MADHD group, this difference was significant (MADHD: t(16)=2.38, p=0.030, d=0.87 +L; ADHD: t(25)=1.86, p=0.074, d=0.71 +m). No interaction between the *ADHD subtype* and the subgroups assigned to the different *training level* was observed in any attention network. Hence, we considered that factor *ADHD subtype* plays only a marginal role in our samples and we focused the model with the values of the attention network effects as a function of factors *group*×*training level*×*session*×*attentionnetwork*. We tested the null hypothesis of homoskedasticity and no heteroscedasticity was found in our data and model (Breusch–Pagan test BP=38.936, df=35, p=0.30). The four-way ANOVA corresponding to our model showed significant main effects of factors *group* (F(2,594)=5.65, p < 0.01, $\omega_{p}^{2}=0.01$ +s), *session* (F(1,594)=4.82, p < 0.05, $\omega_{p}^{2}=0.006$ +si), *attentionnetwork* (F(2,594)=482.02, p < 0.001, $\omega_{p}^{2}=0.60$ +L) and a significant *session*×*attentionnetwork* two-way interaction (F(2,594)=8.70, p < 0.001, $\omega_{p}^{2}=0.02$ +s).

**Figure 5:**
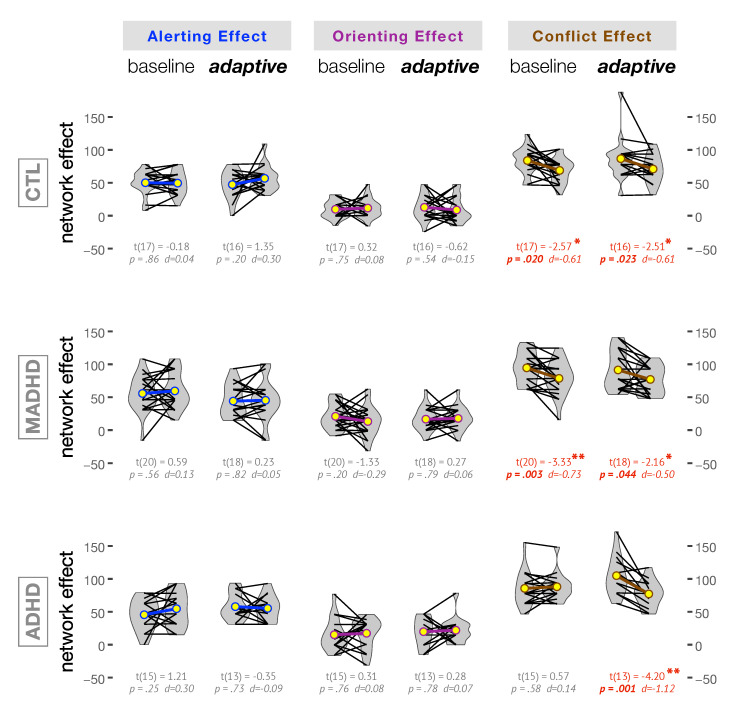
Attention network effects within groups before and after training. The yellow circles in the violin plots correspond to the average values of the corresponding network effects. The *p*-values and Cohen’s *d* effect sizes are reported for paired Student’s *t* tests. Notice that WMT affected only the conflict network. In particular, for ADHD only the *adaptive* training provoked a large and significant effect. Groups: **CTL**: control subjects; **MADHD**: patients **with** medication; **ADHD**: patients **without medication**.

Figure 5 illustrates how training with *baseline* and *adaptive* level affected each group for alerting, orienting, and conflict networks. Within groups comparisons were carried out with Student’s *t* test and corresponding Cohen’s *d* effect size are reported in Figure 5. No effect of training level was observed for *Alerting Effect* and *Orienting Effect* in any group. In controls, the average *Conflict Effect* was significantly decreased after *baseline* and *adaptive* training with moderate effect size (t(17)=2.57, p < 0.05, d=0.61 +m and t(16)=2.51, p < 0.05, d=0.61 +m, respectively). Both training modes affected the average *Conflict Effect* in medicated ADHD patients (t(20)=3.33, p < 0.01, d=0.73 +m and t(18)=2.16, p < 0.05, d=0.50 +m after *baseline* and *adaptive* mode, respectively). On the contrary, in the ADHD *without medication* only the *adaptive* level of training provoked a very large and significant effect (t(13)=4.20, p < 0.01, d=1.12 +L). It is important to note that a decrease in the *Conflict Effect* corresponds to an improvement in the executive control network. In agreement and as a confirmation of this important finding, notice that Table 3 showed only significant differences in RTs before and after *adaptive* level of training for ADHD *without medication*. This is important because the *Conflict Effect* is computed after the RTs following *congruent* and *incongruent* targets (Table 3).

We also analyzed the changes in each attention network as a function of the severity of the symptoms measured by the Conners’ Adult ADHD Rating Scales, i.e., a dimensional analysis irrespective of the group of participants. The only significant correlation was observed between the CAARS:B *DSM-IV Hyperactive-Impulsive Symptoms Subscale* and the *Conflict Effect* after *baseline* training (Spearman’s rank correlation ρ=−0.33, p=0.015 and F(1,52)=5.17, p=0.027, $\omega_{p}^{2}=0.07$ +m). The negative sign means that the lesser the severity of the hyperactive-impulsive symptoms the larger the improvement in the *Conflict Effect*.

### 3.6. Ex-Gaussian Distributional Model of RTs

For each participant (n=105), we computed the three ex-Gaussian parameters *mu* (μ), *sigma* (σ), and *tau* (τ) from the distribution of individual RTs. The parameters *mu* and *sigma* correspond to the estimated mean and standard deviation of the Gaussian portion of the RT distribution. Hence, parameter *sigma* is a good estimate of the RT variability. The parameter *tau* corresponds to an exponential decay parameter associated with the skewness of the tail of the RT distribution. At first, we considered a dimensional analysis of these parameters with the CAARS symptom subscales as factors in one-way ANOVAs and the corresponding correlation coefficients. Studentized Breusch–Pagan tests showed that the values were homoscedastic and we used standard ANOVAs and Pearson’s *r* rank correlation coefficients. Parameters *mu* and *sigma* were positively correlated with the inattentive symptoms score (CAARS:A) (r=0.25, F(1,102)=6.87, p=0.010, $\omega_{p}^{2}=0.05$ +s and r=0.21, F(1,102)=4.92, p=0.029, $\omega_{p}^{2}=0.04$ +s, respectively). No significant correlations was observed between any parameter with the hyperactive-impulsive symptoms score (CAARS:B). Parameter *sigma* was the only one positively correlated with the total symptoms score (CAARS:C) (r=0.19, F(1,102)=3.95, p=0.049, $\omega_{p}^{2}=0.03$ +s). Very significant correlations were observed between the parameters *mu* and *sigma* and the ‘ADHD Index’ (i.e., the normalized T-score of CAARS) (r=0.32, F(1,102)=11.43, p=0.001, $\omega_{p}^{2}=0.09$ +m and r=0.29, F(1,102)=9.51, p=0.003, $\omega_{p}^{2}=0.09$ +m, respectively). Secondly, for each participant, we analyzed the differences between corresponding *mu*, *sigma* and *tau* values computed *after* and *before* the WM training (i.e., Δμ, Δσ and Δτ) with the CAARS symptom subscales as factors in one-way ANOVAs and the corresponding correlation coefficients. Only one significant correlation was observed, between Δτ and the hyperactive-impulsive symptoms score (CAARS:B) after *baseline* training (r=0.35, F(1,52)=7.25, p=0.009, $\omega_{p}^{2}=0.10$ +m). It is interesting to note that no parameters among Δμ, Δσ, and Δτ correlated with any CAARS symptom subscales after *adaptive* training.

We carried out the categorical analysis of the ex-Gaussian parameters in the same way as described for RT, with a model depending on three factors: *patients’ group* × *session* × *training level*. With this model, the parameters *mu*, *sigma* and *tau* were homoscedastic and variances were homogeneous on Levene’s Test. For Ex-Gaussian parameter *mu*, the three-way ANOVA corresponding to our model showed significant main effects of factors *group* (F(2,198)=4.908, p < 0.01, $\omega_{p}^{2}=0.04$ +s), *session* (F(1,198)=10.740, p=0.001, $\omega_{p}^{2}=0.05\;+s$) and a *group* × *training level* two-way interaction just below the threshold of significance (F(2,198)=3.168, p=0.044, $\omega_{p}^{2}=0.02$ +s). For ex-Gaussian parameter *sigma*, the three-way ANOVA yielded the same effects observed for *mu*, i.e., main effects for *group* (F(2,198)=6.416, p=0.002, $\omega_{p}^{2}=0.05$ +s), *session* (F(1,198)=6.390, p=0.012, $\omega_{p}^{2}\;=\;0.03$ +s) and a significant *group* × *training level* two-way interaction (F(2,198)=4.409, p=0.013, $\omega_{p}^{2}=0.03$ +s). No significant effects were observed for parameter *tau*. The values of *mu* were always normally distributed and we used Student *t*-tests for within group and between-groups comparisons and Cohen’s *d* for the effect size. The values of *tau* were never normally distributed and we used the Wilcoxon Signed-Rank test for within group and the Mann–Whitney test for between groups comparisons and *r* value, between 0 and 1, for the effect size. For the values of *sigma*, most distributions were normally distributed and we used the appropriate test following the outcome of the normality test. The values of all ex-Gaussian parameters of RT distributions in all groups of participants and for all experimental conditions, are presented in Table 5. Notice that RT variability associated to parameter σ was significantly reduced after WMT only in the ADHD without medication in the *baseline* condition.

For each group of participants, we have eventually analyzed the effect of the training level during WMT with any significant regression and correlation between the variation of the ex-Gaussian parameters Δμ, Δσ and Δτ and the variation of the attention network effects. Depending on the outcome of the respective normality tests we used either Pearson or Spearman rank correlations. In controls, we observed a decrease in Δσ correlated with an improvement of the *Alerting Effect* after *adaptive* training (r=−0.33, F(1,15)=4.80, p=0.045, $\omega_{p}^{2}=0.18$ +L). In medicated ADHD participants, we observed significant correlations only after *baseline* training and with an improvement of the *Conflict Effect*, with a decrease in Δσ (r=−0.74, F(1,19)=4.59, p=0.045, $\omega_{p}^{2}=0.15$ +L) and with a decrease in Δτ (r=−0.69, F(1,19)=9.99, p=0.005, $\omega_{p}^{2}=0.30$ +L). In ADHD patients without medication, we observed significant positive correlations only after *adaptive* training. We observed that an improvement of the *Conflict Effect* correlated with an increase in Δμ (r=0.44, F(1,12)=9.45, p=0.010, $\omega_{p}^{2}=0.38$ +L) and an improvement of the *Orienting Effect* correlated with an increase in Δτ (r=0.39, F(1,12)=8.31, p=0.014, $\omega_{p}^{2}=0.34$ +L). It is important to notice that most correlations were associated with an improvement of the *Conflict Effect* (i.e., with a decrease in Δτ and a decrease in Δσ in MADHD after *baseline* training and with an increase in Δμ in ADHD without medication after *adaptive* training).

## 4. Discussion

The overall pattern of RTs to the combination of cues and targets in the Attention Network Test observed in this study showed that for any group of participants the RTs were longer after *No Cue* and *Incongruent* target conditions and the RTs were shorter after *Spatial Cue* and *Congruent* target conditions, in agreement with the well-established literature [22,28,69,70,71,72]. In ADHD patients, ANT was studied in children and adults [73,74,75,76,77,78,79,80]. In general, these studies report that RTs of ADHD patients tend to be longer than controls, but accuracy and variability characterized at several degrees those patients with inattentive symptoms and suggested dysfunctions in the coupling between alerting, orienting, and conflict (executive) networks. Several studies exist aiming at the improvement of executive functions in ADHD patients with a focus either on stimulant medication or cognitive working memory training [81,82,83,84,85], however the present study is the first one including medicated and non-medicated ADHD patients performing ANT before and after working memory training.

### 4.1. ADHD Diagnosed Subtypes

It is worth noting some characteristics in the composition of our patients’ samples. Our final sample of medicated ADHD ($N_{\mathrm{MADHD}}=40$) included mostly patients diagnosed with a combined inattentive/hyperactive subtype of ADHD (28 ADHD-C vs. 10 predominantly inattentive type ADHD-I and 2 undefined subtype). On the contrary, the final sample of ADHD ($N_{\mathrm{ADHD}}=30$) included 16 ADHD-C vs. 12 ADHD-I, 1 predominantly hyperactive/impulsive subtype ADHD-HI and 1 undefined subtype. The commonness of ADHD-C (overall 44 patients) with respect to ADHD-I (overall 22 patients) is in agreement with several literature reports in young adults [86,87,88,89]. We found significant differences between the values of the ADHD-C and ADHD-I patients’ score to the ADHD Self-Report Scale (ASRS) [56], in agreement with other citations [90,91]. We also found significant differences between these ADHD subtypes for the values of CAARS:B (DSM-IV hyperactive-impulsive symptoms) and CAARS:C (DSM-IV ADHD total symptoms), in agreement with previous studies [92,93]. However, the current study is definitely underpowered for a thorough ADHD subtype analysis if we consider also the subtype assignments to the subgroups of training protocol. The purpose of this subsection is to raise the attention on the potential effect of ADHD subtype diagnosis on the interpretation of the results.

The MADHD group was characterized by the prevalence of ADHD-C/ADHD-I (28/10) compared to ADHD (16/12), and it is worth noting that our MADHD group was characterized by average values higher than ADHD for ASRS p < 0.05) and for the ADHD Index p < 0.05). It could be argued that ADHD diagnoses exist on a continuum rather than as separate categories on the assumption of symptom ratings distributions (e.g., CAARS subscales scores) could correct some weaknesses of the DSM categorical criteria. Indeed, the dimensional approach of ADHD severity symptoms for discussion of diagnostic issues has been in the focus of DSM-V following several studies showing inconsistencies to support the discrimination of subtypes of DSM-IV ADHD, in particular ADHD-I and ADHD-C [94,95,96]. We analyzed the correlations between RTs and the four CAARS scores and we showed that the more severe the symptoms the longer the RT, thus providing arguments that ADHD diagnoses is associated with ADHD severity on a continuum. Despite the fact that the main effect of factor *ADHD subtype* was not significant in the RT analyses, we observed that before training MADHD reacted with RTs significantly longer than ADHD (although with a small effect size). In the literature, a study suggested that an ADHD subtype reporting effective fluctuations was characterized by slowed RTs to ANT [78], somehow similar to the finding of another study showing that combined inattentive/hyperactive (ADHD-C) patients, but not primarily inattentive (ADHD-I) patients, were also characterized by slowed RTs [73]. Hence, we cannot discard the possibility that slowed RTs in the MADHD group might be due to the prevalence (75%) of ADHD-C patients in that group, but it is also true that the MADHD group is characterized by higher scores of symptoms severity measured by the ADHD Index. It was also reported in the literature that a subset of ADHD-C patients medicated with stimulants could perform ANT nearly at the level of controls [73]. We tested also for any gender or age main effect for the ASRS and all CAARS-S:SV subscales, but none was significant.

Last but not least the double-blind assignment of patients to the subgroups following a *baseline* or an *adaptive* level of training in WMT resulted in an even representation of ADHD subtypes in the *baseline* and *adaptive* subgroups. The same reasoning might hold for the dimensional analysis. However, we observed only a couple of differences between some CAARS scores of the subgroups assigned to *baseline* and *adaptive* prior to WMT. In the non-medicated ADHD group there was a difference very close to threshold (p=0.0497, r=0.36 +m) for the DSM-IV Hyperactive-Impulsive Symptoms Subscale (CAARS:B) dimension. In controls, a significant difference (p=0.019, r=0.40 +m) was observed was observed for the ADHD index (CAARS:D) dimension. We cannot rule out that these differences might have produced an impact on the final outcome of the WMT training analysis, but in both categorical factorial analysis (for the ADHD subtypes) and dimensional analysis (for the CAARS: scores) the interactions due to the factor *training level* were not significant. We dismiss further discussion of this point, but it was worth mentioning for a thorough evaluation of this study.

### 4.2. RT and RT Variability

In this study, irrespective of the assignment subgroup of training and for median RTs for all stimulus patterns merged together, we observed that controls performed faster than ADHD patients of both patients’ groups together (before training p=0.002 with a small effect size r=0.10; after training p=0.005 with a large effect size r=0.63). However, this observation could be misleading for several reasons. Firstly, RTs of MADHD were longer than ADHD possibly because our MADHD sample was characterized by a different composition of ADHD subtypes and by a different intensity in the severity of symptoms assessed by CAARS. Secondly, the computations based on the median RTs for each stimulus pattern eliminate the effect of the biased distribution of RTs, which is usually characterized by long tails towards long RTs. ADHD patients are characterized as ubiquitously slower and with greater RT variability relative to controls [33,34]. Stimulant medication of ADHD in a Go/NoGo task slowed RT and increased RT variability was attenuated, but remained unaffected by non-stimulant medical and psychosocial interventions [31,33]. A meta-analysis review [33] showed also that slower average processing speed in ADHD was not confirmed after accounting for RT variability, whereas large magnitude RT variability deficits remained after accounting for mean RT.

The ex-Gaussian distribution model was used to model RT and RT variability in ADHD performing several tasks [29,30,31,32], but never yet in the attention network task. In contrast to increased mean RT, the distributional parameter μ (derived from the mean of the Gaussian component of the distribution) did not document a significant slowing in adult ADHD patients. Several studies showed that ADHD were characterized by smaller values of parameter μ than controls [30,32,97,98]. In the dimensional analysis of our study, we found a positive correlation between parameter μ and the intensity of inattentive symptoms and ADHD index rated by the Conners’ Adult ADHD Rating Scales-Self Report subscales (CAARS:A and CAARS:D). Adult ADHD, with minimal differences across the ADHD subtypes, were characterized by increased intra-individual variability throughout the entire RT distribution as indicated by the parameters σ (derived from the standard deviation of the RT distribution) and by a greater proportion of abnormally slow responses associated with parameter τ (i.e., the exponential component which reflects the extreme values) [29,34,36,97,99]. We found that parameter σ correlated positively with the severity of the symptoms rated by all CAARS subscales, with the notable exception of hyperactive-impulsive symptoms (CAARS:B). We did not find any significant correlation between the CAARS ratings and parameter τ before WMT. In our categorical analysis, the ANOVA did not reveal any main effect of factor *group* with either ex-Gaussian parameter, in agreement with another study using choice RT tasks that did not demonstrate a group difference without taking into account the comorbities [100].

### 4.3. Working Memory Training

For controls and MADHD, the outcome of the WMT during one month was significant shorter RTs to ANT with either *baseline* or *adaptive* mode in the Dual *n*-back task. In the case of non-medicated ADHD, the significant effect of WMT was observed only if the training was done in the most cognitive demanding version of the task, that is the *adaptive* Dual *n*-back task. The effect of WMT on RTs was already reported for healthy adults [101], but our study is the first one showing a significant effect in adult ADHD. After training, the analysis of network effects showed that there were no significant changes in both *Alerting Effect* and *Orienting Effect* for all participant groups. The literature reports that *Alerting Effect* is improved by the stimulant medication in patients diagnosed as ADHD-C subtype, but not in ADHD-I subtype [73]. In both patients’ groups, we observed improved *Alerting Effect* in ADHD-C vs. ADHD-I subgroups, but this difference was statistically significant only in MADHD. However, there was no significant difference between the two patients’ groups neither before nor after training. Differences between medicated and non-medicated ADHD patients in the *Alerting Effect* might depend on the kind of medication. In the case of dextroamphetamine, extracellular norepinephrine is much more increased than after dopamine [102,103]. Dextroamphetamine was prescribed to the majority (9/14) of the patients of the study reported in the literature [73]. In our MADHD group, methylphenidate, which affects mainly the dopamine system, was prescribed to all patients. These drugs have a very different mechanism of action [104,105,106,107] and the alerting network involves brain areas activated by the norepinephrine system [26]. Therefore, it is likely that the patients of our MADHD group are less affected by the medication with respect to the values of *Alerting Effect* reported elsewhere [73].

In our WMT, the *baseline* mode of the Dual *n*-back task corresponds to the 1-back, i.e., when participants were required to detect a match with the immediately previous trial. This means that the *baseline* mode is characterized by a rather moderate attentional and cognitive load. Then, it is interesting to notice that WMT produced a little improvement, if any, in the conflict (or executive control) network of the ADHD group without medication. The outcome of the ex-Gaussian analysis was in the same direction, with a much stronger correlation of Δμ with *Conflict Effect* after *adaptive* (p=0.010) than after *baseline* training (p=0.048). Both controls and MADHD showed an improvement of *Conflict Effect* by WMT (Figure 5). In agreement with this finding, the dimensional analysis after *baseline* training showed a significant correlation such that the lesser the severity of the hyperactive-impulsive symptoms (CAARS:B) the larger the improvement in the *Conflict Effect*. In addition, the lesser the severity of these symptoms the smaller the variation in the tail of RT distributions (i.e., Δτ) and the smaller the Δτ the larger the improvement in the *Conflict Effect*. These findings are in agreement with the ex-Gaussian analysis reported for a Stroop task showing that the response conflicts mainly affected the Gaussian components, whereas the task conflicts were more prominent in the exponential component τ [108].

The different outcome of *baseline* mode on *Conflict Effect* between ADHD groups with and without medication might be explained by the effect of the stimulants [73,109]. The conflict (or executive control) network is mainly modulated by the dopamine system and involves brain structures in the prefrontal and anterior cingulate cortex, the anterior insula, and the basal ganglia [110,111,112]. It has been suggested that WMT might change the density of cortical dopamine receptors in the prefrontal cortex [113,114,115]. It is known that the activity of the prefrontal cortex, especially in the right hemisphere, is impaired in adult ADHD patients [116,117,118]. This impairment is likely to be responsible of the deficits in response inhibition and working memory [119,120,121], as suggested in previous studies of ANT with ADHD children [74,122]. Medication by methylphenidate is meant to block the reuptake of dopamine and noradrenaline in the central nervous system and result in increased concentrations of dopamine at the synaptic cleft [123,124]. This may explain why WMT in the *baseline* mode of training did not improve *Conflict Effect* in our participants belonging to the non-medicated ADHD group.

On the contrary, the *adaptive* mode of training produced an unexpected and significant (p < 0.01) improvement of *Conflict Effect* in non-medicated ADHD patients. These patients demonstrated a poor functioning of the conflict network when resolving the conflict generated by the *Incongruent* target stimuli (Figure 4). However, after *adaptive* training, the corresponding ADHD subgroup showed that RTs became significantly shorter even with *Incongruent* stimuli (Table 3). Other studies reported that WMT contributed to reducing ADHD symptoms and reinforcing inhibitory control after computerized WMT [83] and *n*-back training in ADHD children [125]. Therefore, we raise the hypothesis that WMT with high demanding attentional and cognitive load may contribute to improving conflict network performance by means of activation of the dopaminergic pathway in the prefrontal cortex. Then, a training with Dual *n*-back task in the *adaptive* mode carries the potential to reduce adults’ ADHD symptoms. It is important to consider also the role played by motivation. Impairment in response inhibition [126] and motivational dysfunction with a serious sensitivity for immediate rewards [127] are among the most important deficits associated with patients suffering of ADHD. The elevated need of reinforcement in these patients may result in motivational problems during executive tasks and cognitive training when the subject has to repeat the same response over and over again for many trials, making most cognitive training tedious and boring [128]. Cognitive-motivational deficits associated with ADHD are a factor of treatment adherence especially regarding the degree of interest and stimulation of tasks [129]. For these motivational reasons, WMT in *adaptative* condition could trigger subject’s engagement and might be considered more rewarding than in *baseline* condition, especially for the ADHD group without medication.

### 4.4. Limitations and Future Investigations

The results presented here should be considered in light of some limitations. The current study, as any other one with ADHD patients, is influenced by the limited size of the samples and by the heterogeneity in symptoms and executive function deficits observed in the groups of ADHD and MADHD patients. The design of this study corresponds to the clinical practice combining medication and other interventions. The combination between clinical and pharmacological interventions is usually considered as first-line treatment for ADHD [130]. Nevertheless, there is a need of future studies aimed at cost and time-effective multimodal treatments with more effective and adjunctive interventions for ADHD [131] and the mechanisms underlying cognitive and symptoms enhancement [84].

We have focused our study on the effects of WMT on ANT, which led us to give priority to the double-blind assignment of patients to the subgroups trained either with the *baseline* or *adaptive* mode of the Dual *n*-back task. The consequence is that the subgroups were not balanced with respect to patients’ diagnosis as ADHD-C or ADHD-I, but the cofactor *ADHD subtype* did not appear to play a major role in the statistical effect of the interactions computed in our analyses. A theoretical optimal design of the study should include a group of medication-naive participants receiving a placebo, which would allow us to better separate the intrinsic effects of medication. However, such a design is not allowed by ethics committees. Additional randomization of the patients’ assignment in subgroups taking into account motivational factors and ADHD developmental factors in patients’ life course would certainly contribute to better identification of the attention network components liable to be influenced by WMT [36,125,132,133]. Furthermore, patients with ADHD are particularly sensitive to immediate reinforcement [36,134], which makes measurements of attention processes by ANT after WMT difficult to interpret given a lack of ecological validity on the daily functioning of adult ADHD patients [135,136].

The completeness of this study could also be improved. The dimensional analysis has shown that longer RTs were associated with more severe symptoms scored in any of the Conners’ Adult ADHD Rating Scales-Self Report (Screening Version, CAARS-S:SV) subscales, with the notable exception of the ADHD group without medication after *adaptive* training. This result is interesting because it suggests that, in ADHD, the overall decrease in RTs after *adaptive* training (by 26.5±0.6 ms, p < 0.001 with large effect size r=0.64) is likely to be associated with cognitive processes not tested thoroughly by CAARS. Recent studies of adult ADHD patients medicated with atomoxetine [137,138] pointed out that executive functioning in everyday life may be better assessed by means of the questionnaire Behavior Rating Inventory of Executive Function-Adult Version BRIEF−A [139,140]. We recommend to include both CAARS and BRIEF in future studies.

## 5. Conclusions

The Attentional Network Task allows testing the plasticity of brain circuits in ADHD patients in a notable way. The present study demonstrates that working memory training for one month using the Dual *n*-back task training in the *adaptive* mode produced a significant improvement in such *Conflict Effect* of adult ADHD patients irrespective of their medication. The *baseline* mode was insufficient to produce measurable effects in the non-medicated ADHD patients, which may explain previous contradictory reports in the literature with respect to the usefulness of working memory training. Hence, the Dual *n*-back task in the *adaptive* mode offers as a promising candidate for a cognitive remediation of adult ADHD patients without pharmaceutical medication.

## Figures and Tables

**Table 1:** Descriptive statistics (median, mean, and SEM) of participants’ accuracy rates of performance and DSM-IV symptom subscales. The effects of factors *group* and *level* of training were tested by a two-way analysis of variance (ANOVA). The effect size was estimated by $\omega_{p}^{2}$.

	Group:	Controls		MADHD		ADHD		*ANOVA*		*p*-Value	*Effect Size*
	Level:	**Baseline**	**Adaptive**	**Baseline**	**Adaptive**	**Baseline**	**Adaptive**	***Effect***	F	**Pr**(>F)	$\omega_{p}^{2}$
**Sample size (** ***N*** **)**		20	18	22	20	17	17				
*After outlier removal*		18	17	21	19	16	14				
ADHD−C		−	−	16	12	8	8				
ADHD−I		−	−	4	6	8	4				
ADHD−HI		−	−	0	0	0	1				
unknown ADHD subtype		−	−	1	1	0	1				
**ANT Accuracy Rate AR**		98.6%	98.9%	98.2%	98.2%	97.9%	98.2%	group:	1.85	0.16	0.02 +s
*(before WMT)*		98.4 (3.9)	98.4 (4.1)	97.3 (4.9)	98.0 (3.1)	97.5 (6.1)	97.8 (4.4)	level:	0.85	0.36	0.00 +si
								group×level:	0.26	0.78	0.01 +si
**ANT Accuracy Rate AR**		98.3%	98.6%	97.5%	97.9%	98.1%	97.7%	group:	1.06	0.35	0.00 +si
*(after WMT)*		97.6 (4.8)	98.2 (3.9)	97.0 (5.1)	97.5 (3.9)	97.9 (4.1)	97.4 (4.9)	level:	0.48	0.49	0.00 +si
								group×level:	0.89	0.42	0.00 +si
**Adult ADHD**		47.5	47.0	68.0	62.0	60.0	55.0	group:	25.65	<0.001 ***	0.32 +L
**self−report Scale(ASRS)**		48.3 (3.0)	45.7 (2.2)	67.6 (2.4)	63.6 (3.0)	59.9 (2.9)	55.6 (2.3)	level:	2.74	0.10	0.02 +s
								group×level:	0.05	0.95	−0.02 +si
***Inattentive symptoms***		56.0	51.0	79.5	76.0	75.0	74.0	group:	51.9	<0.001 ***	0.49 +L
		56.6 (2.8)	49.8 (2.0)	79.1 (1.8)	74.7 (2.2)	73.6 (3.2)	70.8 (3.1)	level:	5.35	<0.05 *	0.04 +s
								group×level:	0.29	0.75	0.01 +si
***Hyperactive−impulsive***		47.5	41.0	67.0	59.0	69.0	59.0	group:‡	47.3	<0.001 ***	0.87 +L
***symptoms***		47.3 (2.1)	42.7 (2.1)	64.3 (4.0)	57.7 (3.8)	65.5 (2.5)	57.4 (2.8)	level:‡	6.36	<0.05 *	0.29 +s
								group×level:‡	0.23	0.89	−
***Total ADHD***		58.0	44.0	78.0	69.0	78.0	64.5	group:	47.2	<0.001 ***	0.47 +L
***symptoms***		52.9 (2.4)	45.8 (2.0)	75.5 (2.6)	69.4 (3.3)	73.9 (3.1)	67.6 (2.3)	level:	8.86	<0.01 **	0.07 +m
								group×level:	0.02	0.98	−0.02 +si
***ADHD Index***		49.0	45.0	66.5	60.0	56.0	57.0	group:	29.3	<0.001 ***	0.35 +L
		49.5 (1.8)	45.5 (1.9)	66.4 (2.6)	60.8 (2.2)	56.9 (2.0)	58.1 (2.2)	level:	3.02	0.09	0.02 +s
								group×level:	1.25	0.29	0.00 +si

‡: due to significant heteroscedasticity, test with robust two-way ANOVA for trimmed means, instead of standard ANOVA. Significance codes of p-values. *: *p* < 0.05; **: *p* < 0.01; ***: *p* < 0.001. Magnitude of effect sizes. +si: statistically insignificant; +s: small; +m: medium; +L: large.

**Table 2:** Descriptive statistics for robust linear regression y=a+bx (a: intercept, b: slope) and for Spearman’s rank correlation (rho and *p*-value) between reaction times and four CAARS scores associated with symptoms severity. Analysis is reported for all participants merged into the same sample, assuming that the correlation depends only on the scored symptoms and for each participants’ group separately. NS: not significant.

			Baseline Training					Adaptive Training			
CAARS Scale			Robust Regression		Correlation			Robust Regression		Correlation	
			*Intercept*	*Slope*	ρ	*p*-Value		*Intercept*	*Slope*	ρ	*p*-Value
CAARS:A ***DSM-IV Inattentive Symptoms Subscale***											
ALL participants	BEFORE WMT		417.31	0.825	0.139	<0.001		425.85	0.764	0.132	<0.001
	**AFTER** WMT		421.30	0.766	0.124	<0.001		426.10	0.655	0.112	<0.001
Controls	BEFORE WMT		417.27	0.927	0.160	<0.001		420.69	0.887	0.153	<0.001
	**AFTER** WMT		417.86	0.856	0.149	<0.001		417.90	0.811	0.132	<0.001
ADHD patients	BEFORE WMT		407.09	0.914	0.153	<0.001		433.95	0.617	0.113	<0.001
**with** medication	**AFTER** WMT		423.04	0.702	0.114	<0.001		415.32	0.835	0.140	<0.001
ADHD patients	BEFORE WMT		428.64	0.632	0.104	<0.001		419.28	0.845	0.132	<0.001
*without* medication	**AFTER** WMT		424.34	0.728	0.102	<0.001		450.32	0.222	0.040	0.0149
CAARS:B ***DSM-IV Hyperactive-Impulsive Symptoms Subscale***											
ALL participants	BEFORE WMT		440.93	0.580	0.086	<0.001		438.82	0.688	0.103	<0.001
	**AFTER** WMT		448.06	0.457	0.061	<0.001		444.13	0.466	0.067	<0.001
Controls	BEFORE WMT		429.90	0.881	0.133	<0.001		442.87	0.661	0.104	<0.001
	**AFTER** WMT		442.26	0.598	0.088	<0.001		424.16	0.860	0.131	<0.001
ADHD patients	BEFORE WMT		437.04	0.587	0.077	<0.001		443.81	0.565	0.082	<0.001
**with** medication	**AFTER** WMT		457.93	0.230	0.033	0.0167		446.13	0.457	0.063	<0.001
ADHD patients	BEFORE WMT		457.24	0.257	0.036	0.0208		427.39	0.885	0.123	<0.001
*without* medication	**AFTER** WMT		442.18	0.580	0.065	<0.001		464.64	0.011	0.017	**0.3137 NS**
CAARS:C ***DSM-IV Total ADHD Symptoms Subscale***											
ALL participants	BEFORE WMT		422.69	0.790	0.124	<0.001		425.80	0.804	0.132	<0.001
	**AFTER** WMT		432.14	0.641	0.104	<0.001		434.41	0.557	0.101	<0.001
Controls	BEFORE WMT		414.69	1.013	0.163	<0.001		421.48	0.913	0.150	<0.001
	**AFTER** WMT		429.25	0.721	0.138	<0.001		419.28	0.825	0.156	<0.001
ADHD patients	BEFORE WMT		415.98	0.836	0.125	<0.001		434.76	0.638	0.104	<0.001
**with** medication	**AFTER** WMT		437.65	0.516	0.082	<0.001		428.71	0.669	0.109	<0.001
ADHD patients	BEFORE WMT		440.01	0.493	0.075	<0.001		417.04	0.927	0.139	<0.001
*without* medication	**AFTER** WMT		429.67	0.690	0.088	<0.001		459.67	0.087	0.010	**0.5267 NS**
CAARS:D ***‘ADHD Index’, the normalized T-score of CAARS***											
ALL participants	BEFORE WMT		406.53	1.173	0.141	<0.001		414.51	1.115	0.131	<0.001
	**AFTER** WMT		412.53	1.081	0.133	<0.001		417.72	0.932	0.129	<0.001
Controls	BEFORE WMT		379.32	1.786	0.188	<0.001		410.5	1.249	0.132	<0.001
	**AFTER** WMT		378.32	1.738	0.200	<0.001		376.89	1.714	0.219	<0.001
ADHD patients	BEFORE WMT		404.90	1.121	0.147	<0.001		417.62	1.014	0.131	<0.001
**with** medication	**AFTER** WMT		437.30	0.589	0.084	<0.001		415.74	0.985	0.125	<0.001
ADHD patients	BEFORE WMT		430.91	0.717	0.084	<0.001		410.57	1.177	0.134	<0.001
*without* medication	**AFTER** WMT		420.13	0.958	0.109	<0.001		463.67	0.028	0.015	**0.3777 NS**

**Table 3:** Descriptive statistics (median, mean, and SEM) of reaction times for each group of participants and for any cue type (pooled together) as a function of *Congruent* and *Incongruent* targets. Comparisons between levels of training (Mann–Whitney test) and between before and after working memory training (WMT) within each group (Wilcoxon Signed-Rank test). For each test the corresponding *p*-values and effect size (*r*) are reported in the table.

	Target:	Congruent		Mann–Whitney Test		Incongruent		Mann–Whitney Test	
Reaction Times (ms)	Levtel:	Baseline	Adaptive	*(* *Between Levels* *)*		Baseline	Adaptive	*(Between Levels)*	
				*p*-Value	*r*			*p*-Value	*r*
	BEFORE WMT	437.3	453.0	0.08	0.15 +s	531.0	531.5	0.09	0.14 +s
**Controls**		440.4 (6.6)	457.0 (7.0)			524.3 (7.2)	546.3 (7.8)		
	**AFTER** WMT	421.5	438.0	0.15	0.12 +s	500.0	507.8	0.12	0.13 +s
		427.9 (5.1)	443.5 (7.1)			497.1 (5.9)	513.3 (7.1)		
Wilcoxon Signed-Rank test	*p*-value	<0.01 **	<0.01 **			<0.001 ***	<0.001 ***		
*(within group)*	effect size*r*	0.32 +m	0.37 +m			0.63 +L	0.57 +L		
	BEFORE WMT	453.0	480.3	<0.05 *	0.19 +s	547.0	550.8	0.05	0.15 +s
ADHD patients		458.6 (6.3)	490.2 (8.9)			548.8 (6.8)	582.7 (10.0)		
**with** medication	**AFTER** WMT	437.3	438.0	0.39	0.07 +si	515.0	519.8	0.39	0.07 +si
		445.4 (7.0)	450.8 (6.2)			520.7 (7.2)	528.2 (6.5)		
Wilcoxon Signed-Rank test	*p*-value	<0.001 ***	<0.001 ***			<0.001 ***	<0.001 ***		
*(within group)*	effect size*r*	0.43 +m	0.57 +L			0.55 +L	0.68 +L		
	BEFORE WMT	453.0	429.5	<0.05 *	0.19 +s	546.8	543.3	0.72	0.03 +si
ADHD patients		460.1 (6.9)	439.1 (6.1)			546.6 (6.7)	545.0 (8.4)		
*without* medication	**AFTER** WMT	445.5	414.3	<0.01 **	0.29 +s	531.8	496.3	<0.001 ***	0.38 +m
		451.1 (5.8)	422.7 (6.2)			536.7 (5.7)	497.3 (6.0)		
Wilcoxon Signed-Rank test	*p*-value	0.34	<0.001 ***			0.12	<0.001 ***		
*(within group)*	effect size*r*	0.12 +s	0.50 +L			0.04 +si	0.83 +L		

Significance codes of *p*-values. *: p < 0.05; **: p < 0.01; ***: p < 0.001. Magnitude of effect sizes *r*. +si: statistically insignificant; +s: small; +m: medium; +L: large.

**Table 4:** Descriptive statistics (median, mean, and SEM) of reaction times for each group of participants and for any target type (pooled together) as a function of *Spatial Cue* and *No Cue*. Comparisons between levels of training (Mann–Whitney test) and between before and after working memory training (WMT) within each group (Wilcoxon Signed-Rank test). For each test the corresponding *p*-values and effect size (*r*) are reported in the table.

	Cue:	Spatial Cue		Mann–Whitney Test		No Cue		Mann–Whitney Test	
Reaction Times (ms)	level:	Baseline	Adaptive	*(Between Levels)*		Baseline	Adaptive	*(Between Levels)*	
				*p*-Value	*r*			*p*-Value	*r*
	BEFORE WMT	437.5	453.0	0.12	0.15 +s	484.8	516.0	0.19	0.13 +s
**Controls**		447.2 (8.7)	466.6 (9.2)			504.3 (8.6)	519.7 (8.8)		
	**AFTER** WMT	410.8	438.0	0.13	0.15 +s	484.0	500.0	0.08	0.17 +s
		428.2 (6.6)	446.5 (8.5)			485.8 (6.3)	504.0 (7.9)		
Wilcoxon Signed-Rank test	*p*-value	<0.01 **	<0.01 **			<0.01 **	<0.05 *		
*(within group)*	effect size*r*	0.46 +m	0.49 +m			0.37 +m	0.35 +m		
	BEFORE WMT	461.0	484.0%	0.06	0.17 +s	531.0	547.0	0.07	0.17 +s
ADHD patients		468.9 (8.5)	501.2 (11.3)			523.6 (8.6)	557.7 (12.3)		
**with** medication	**AFTER** WMT	453.0	453.0	0.37	0.08 +si	500.0	500.0	0.73	0.03 +si
		450.4 (7.8)	459.6 (7.5)			506.1 (8.6)	509.7 (8.7)		
Wilcoxon Signed-Rank test	*p*-value	<0.001 ***	<0.001 ***			<0.01 **	<0.001 ***		
*(within group)*	effect size*r*	0.47 +m	0.56 +L			0.42 +m	0.61 +L		
	BEFORE WMT	453.0	437.0	0.10	0.17 +s	511.5	500.0	0.85	0.02 +si
ADHD patients		464.6 (8.8)	443.7 (8.3)			514.3 (8.8)	513.6 (10.9)		
*without* medication	**AFTER** WMT	438.0	418.2	<0.01 **	0.29 +s	515.0	492.5	<0.05 *	0.27 +s
		451.7 (7.1)	421.0 (7.5)			516.8 (6.8)	485.7 (7.2)		
Wilcoxon Signed-Rank test	*p*-value	0.18	<0.001 ***			0.95	<0.001 ***		
*(within group)*	effect size*r*	0.19 +s	0.67 +L			0.00 +si	0.63 +L		

Significance codes of *p*-values. *: p < 0.05; **: p < 0.01; ***: p < 0.001. Magnitude of effect sizes *r*. +si: statistically insignificant; +s: small; +m: medium; +L: large.

**Table 5:** Descriptive statistics (median, mean, and SEM) of ex-Gaussian parameters (*mu*, *sigma*, *tau*) for each group of participants. Comparisons between levels of training and between before- and after-training within each group are reported with the corresponding *p*-values and effect sizes Cohen’s *d* or non-parametric *r* computed following the outcome of the normality tests.

		mu (μ)		*Between*	sigma (σ)		*Between*	tau (τ)		*Between*
	Level:	Baseline	Adaptive	*Groups*	Baseline	Adaptive	*Groups*	Baseline	Adaptive	*Groups*
				*p*, eff.size			*p*, eff.size			*p*, eff.size
**Controls**	BEFORE WMT	395.7	424.4	0.29	45.6	49.7	0. 40	64.9	79.7	0.42
		404.7 (10.3)	419.7 (9.5)	d:0.36 +s	45.9 (3.4)	49.8 (3.2)	d:0.29 +s	75.0 (6.9)	77.9 (6.7)	r:0.14 +s
	**AFTER** WMT	387.7	393.1	0.44	44.2	37.7	0.83s	64.5	58.5	0.88
		391.8 (7.7)	400.4 (7.9)	d:0.26 +s	42.8 (2.7)	45.0 (3.9)	r:0.04 +si	65.8 (3.4)	77.5 (9.9)	r:0.04 +si
*within group:*	*p*-value	0.021 *	0.012 *		0.39	0.28		0.07	0.82	
	effect size	d:0.60 +m	d:0.68 +m		r:0.21 +s	d:0.27 +s		r:0.44 +m	r:0.06 +si	
ADHD patients	BEFORE WMT	415.0	444.0	0.14	55.7	62.9	0.25	70.7	71.3	0.67
		420.0 (10.6)	445.8 (13.6)	d:0.48 +s	54.2 (4.0)	60.8 (4.0)	d:0.37 +s	80.2 (6.7)	89.8 (10.6)	r:0.07 +si
**with** medication	**AFTER** WMT	396.8	418.8	0.49	45.6	55.4	0.19s	64.7	63.2	0.35
		404.4 (9.9)	413.3 (7.8)	d:0.22 +s	48.4 (3.2)	57.8 (5.3)		77.0 (6.9)	73.6 (10.9)	r:0.15 +s
*within group:*	*p*-value	0.033 *	0.011 *		0.28	0.13		0.66	0.036 *	
	effect size	d:0.50 +m	d:0.65 +m		d:0.24 +s	r:0.35 +m		r:0.10 +s	r:0.48 +m	
ADHD patients	BEFORE WMT	418.1	393.9	0.08	54.4	50.8	0.038 *	73.7	83.5	0.38
		416.5 (7.8)	398.1 (6.7)	d:0.65 +m	58.0 (2.9)	47.7 (3.7)	d:0.81 +L	79.5 (7.1)	87.3 (8.6)	r:0.17 +s
**without medication**	**AFTER** WMT	395.1	392.3	0.25	43.9	45.5	0.34s	90.7	62.2	0.015 *
		401.1 (7.3)	389.0 (7.3)	d:0.43 +s	46.8 (4.5)	40.2 (5.0)	d:0.36 +s	90.0 (6.8)	67.7 (5.2)	r:0.44 +m
*within group:*	*p*-value	0.13	0.040 *		0.033 *	0.06		0.13	0.017 *	
	effect size	d:0.41 +s	d:0.61 +m		d:0.59 +m	d:0.54 +m		r:0.39 +m	r:0.63 +L	

Significance codes of *p*-values. *: p < 0.05; **: p < 0.01; ***: p < 0.001. Magnitude of effect sizes *r*. +si: statistically insignificant; +s: small; +m: medium; +L: large.
